# Segregation of Central Ventricular Conduction System Lineages in Early SMA+ Cardiomyocytes Occurs Prior to Heart Tube Formation

**DOI:** 10.3390/jcdd3010002

**Published:** 2016-01-21

**Authors:** Caroline Choquet, Laetitia Marcadet, Sabrina Beyer, Robert G. Kelly, Lucile Miquerol

**Affiliations:** Aix-Marseille Université, CNRS UMR 7288, Developmental Biology Institute of Marseille, Campus de Luminy Case 907, 13288 Marseille Cedex 9, France; caroline.choquet@univ-amu.fr (C.C.); laetitia.marcadet@gmail.com (L.M.); sabadri@orange.fr (S.B.); robert.kelly@univ-amu.fr (R.G.K.)

**Keywords:** cardiac conduction system, fate mapping, clonal analysis, heart tube, smooth muscle actin, cardiac development, atrioventricular bundle, bundle branches

## Abstract

The cardiac conduction system (CCS) transmits electrical activity from the atria to the ventricles to coordinate heartbeats. Atrioventricular conduction diseases are often associated with defects in the central ventricular conduction system comprising the atrioventricular bundle (AVB) and right and left branches (BBs). Conducting and contractile working myocytes share common cardiomyogenic progenitors, however the time at which the CCS lineage becomes specified is unclear. In order to study the fate and the contribution to the CCS of cardiomyocytes during early heart tube formation, we performed a genetic lineage analysis using a *Sma-CreERT2* mouse line. Lineage tracing experiments reveal a sequential contribution of early *Sma* expressing cardiomyocytes to different cardiac compartments, labeling at embryonic day (E) 7.5 giving rise to the interventricular septum and apical left ventricular myocardium. Early *Sma* expressing cardiomyocytes contribute to the AVB, BBs and left ventricular Purkinje fibers. Clonal analysis using the *R26-confetti* reporter mouse crossed with *Sma-CreERT2* demonstrates that early *Sma* expressing cardiomyocytes include cells exclusively fated to give rise to the AVB. In contrast, lineage segregation is still ongoing for the BBs at E7.5. Overall this study highlights the early segregation of the central ventricular conduction system lineage within cardiomyocytes at the onset of heart tube formation.

## 1. Introduction

The cardiac conduction system (CCS) generates and propagates electrical impulses to coordinate atrial and ventricular contraction. The pacemaker or sinoatrial node (SAN) initiates electrical activity that transits through the atrioventricular node (AVN), the unique electrical pathway between atria and ventricles. Subsequently, the ventricular conduction system (VCS), comprised of central components including the atrioventricular or His-Bundle (AVB) and bundle branches (BB) and the peripheral Purkinje Fiber (PF) network, leads to the rapid propagation of electrical activity to trigger apex to base ventricular contraction [1]. Defects in the propagation of electrical activity are a leading cause of conduction defects and atrioventricular (AV) blocks [2]. Defining the developmental origin of the AV conduction pathway is an indispensable step towards understanding the etiology of conduction diseases.

In the mouse embryo, the first heartbeats are detected at embryonic day (E) 8.5 as peristaltic contractions of the primary cardiac tube, which are replaced by sequential contractions of the atria and ventricles at E9.5 as cardiac chambers form [3]. At this stage, the primordium of the AV conduction pathway is distinct in the inner dorsal wall of the atrioventricular canal (AVC) and becomes increasingly compact as development proceeds to form the AVN [4]. At the same time, a group of cells along the ridge of the interventricular septum (IVS) form the primary ring and have been described as the progenitors of the AVB and BB [5]. However, it is unclear from which progenitors adult VCS components originate during embryogenesis and precisely when central and peripheral VCS lineages are specified.

The heart derives from anterior splanchnic mesoderm during gastrulation, within which two cardiac lineages can be distinguished, corresponding to the first and the second heart fields (FHF and SHF) [6,7]. These two populations of cardiac progenitor cells participate differently and sequentially to the embryonic heart. The FHF forms the primary cardiac tube at E8.5 and gives rise to the left ventricle and left part of the interventricular septum, whereas the SHF is added later from pharyngeal mesoderm and gives rise to the right ventricle, including the right part of the inter-ventricular septum and outflow tract myocardium [8,9]. Recently, genetic lineage analyses in mouse embryos, using regulatory sequences specific to the FHF and the SHF, have provided new insights into the origin of the VCS [10,11].

A clonal retrospective analysis combined with a *Connexin Cx40-GFP* reporter mouse line revealed that the VCS is comprised of cells derived from two progenitor cell lineages that contribute to either the right Purkinje fiber network or left BB, likely corresponding to contributions of first and second heart field progenitor cells [12]. However, both lineages were found to participate in the formation of the AV conduction system including the AVN, AVB, and right BB. The gene encoding the hyperpolarization-activated cation-selective nucleotide-gated channel 4 (Hcn4), required for the pacemaker function of conducting cardiomyocytes, is expressed early in the cardiac crescent and primary heart tube where *Hcn4* expression overlaps with FHF (Nkx2.5 and Tbx5) but not SHF markers (Isl1) [13]. Thus, *Hcn4* has been described as an FHF marker in early embryo. From E16.5 to adult heart *Hcn4* expression is restricted to the CCS. Genetic lineage tracing analyses using tamoxifen-inducible *Hcn4-CreERT2* mice show that *Hcn4* expressing cells at early stages of development give rise to FHF-derived structures in the heart, including a limited contribution to the AVB [13,14]. The contribution of the *Hcn4* expressing cells to the CCS varies depending on the time of Cre induction, the entire CCS only being labelled after induction at late fetal stages. These genetic tracing analyses suggest that the central VCS is derived mainly from the FHF, although the timing of the lineage segregation remains unclear.

In order to clarify the stage at which FHF progenitor cells become specified to the AV conduction system, we performed a genetic lineage analysis of early cardiomyocytes using a *Sma-CreERT2* mouse line [15]. Smooth muscle actin (SMA) is an isoform of mammalian actin expressed in smooth muscle cells; however, during embryonic development, SMA is expressed as early as E7.5 in the cardiac crescent and its expression persists in cardiomyocytes until birth [16]. The expression of SMA precedes that of most cardiac markers, suggesting that SMA marks the onset of myocardial differentiation [17,18]. Indeed, the use of *Sma-CreERT2* and *R26R-lacZ* transgenic mice reveals that SMA is expressed in differentiating cardiomyocytes during early heart development [15]. Here we show that cardiomyocytes expressing *SMA* at E7.5 contribute to the linear heart tube and later to FHF-derived parts of the heart. Our lineage analysis reveals a sequential contribution of early SMA+ cardiomyocytes to the FHF and SHF-derived parts of the heart to consecutively build the interventricular region and the left ventricle, followed by the atria, the right ventricle and the atrioventricular canal and finally the OFT and SV. Using a *Cx40-GFP* allele we show that early SMA expressing cardiomyocytes contribute to the CCS at E18 and P7. Furthermore, clonal analysis using the *R26-confetti* reporter mouse crossed with *Sma-CreERT2*, highlights the early segregation of the central VCS lineage within SMA positive cells.

## 2. Experimental Section

### 2.1. Transgenic Lines and Tamoxifen Injection

The *Cx40-GFP*, *Sma-CreERT2*, *R26R-LacZ*, *R26R-YFP*, and *R26R-confetti* mouse lines have been previously reported and mice and embryos were genotyped as described previously [15,19,20,21,22].

For lineage analysis, Sma-CreERT2 males were crossed with R26R females and 4-Hydroxytamoxifen (4-OHT) was injected intraperitoneally to pregnant female at different timepoints (E7.5 and E8.5). 4-OHT (Sigma, Saint-Louis, MO, USA, H7904) was dissolved at a concentration of 20 mg/mL in 100% ethanol, then diluted in Cremophor^®^ EL (Sigma) to 10 mg/mL. Before injection, 4-OHT was diluted in 1X PBS to 3 mg/mL and 200 µL of this solution was injected intraperitoneally into pregnant females. For prospective clonal analysis, *Sma-CreERT2* males were crossed with *R26-confetti* females and the dose of 4-OHT injected was reduced to 100 to 400 µg per female.

### 2.2. Antibodies and Immunofluorescence

Antibodies used in this study are specific to Nkx2-5 (Sc8697 Santa-Cruz, Dallas, TX, USA), α-smooth muscle actin (Sigma, F3777), rabbit anti-β-galactosidase (Cappel, MP Biomedicals, Aurora, OH, USA), GFP (AbD Serotec, Bio-Rad, Hercules, CA, USA), Tbx5 (Sc17866 Santa Cruz, Dallas, TX, USA), Hcn4 (AB5808 Millipore, Darmstadt, Germany), and Contactin-2 (AF1714 R&D Systems, Minneapolis, MN, USA).

For whole-mount immunofluorescence, embryonic hearts were dissected and fixed in 2% paraformaldehyde for 2–6 h at 4 °C, washed in PBS, permeabilized in PBS 1X/0.5% Triton X100 for 1 h and incubated for 3 h in saturation buffer (PBS 1X; 3% BSA; 0.1% Triton X100). The primary antibodies were incubated in saturation buffer for overnight at 4 °C. Secondary antibodies coupled to fluorescent molecules were incubated in saturation buffer and after washes, hearts were observed under a Zeiss Lumar stereomicroscope V12 or Zeiss LSM780 confocal microscope. For immunofluorescence on cryostat sections, we performed the same procedure as described previously [19].

### 2.3. X-gal Staining

For X-gal staining of β-galactosidase activity, whole-mount embryos or hearts were fixed in 2% paraformaldehyde for 30 min at 4 °C, washed in PBS and then incubated with X-gal solution (25 mg/mL X-gal in dimethylformamide diluted in 2 mM MgCl_2_, 0.01% deoxycholate, 0.02% Nonidet P40, 0.21% potassium ferrocyanide (Sigma P9387), 0.164% potassium ferricyanide (Sigma, P8131), and PBS overnight at 37 °C. After washes in PBS, tissues were postfixed for 1 h in 4% paraformaldehyde.

### 2.4. Clonal Analysis

*SMA-CreERT2* mice were crossed with *Rosa-confetti* reporter mice and low dose of 4-OH tamoxifen injections were performed at E7.5 to induce rare recombination events, generating well-separated clones. At P7, hearts were dissected and fixed and fluorescent clones were visualized under Zeiss Lumar stereomicroscope V12 using appropriate filters to detect RFP, GFP/YFP, and CFP. Hearts were then processed for cryosections and stained with an anti-Contactin-2 antibody as described above. Individual clusters were identified on serial sections and classified by color and localization. A single clone corresponds to a cluster composed of cells of only one color (RFP, GFP, YFP, or CFP) that is distributed in consecutive sections. Clones with 100% or a restricted number of Cntn-2-positive cells were classified as conducting or mixed, respectively, while those with no Cntn-2-positive cells were classified as working.

## 3. Results

### 3.1. SMA+ Cells in the Early Heart Tube Contribute to the Interventricular Septum

**Figure 1:**
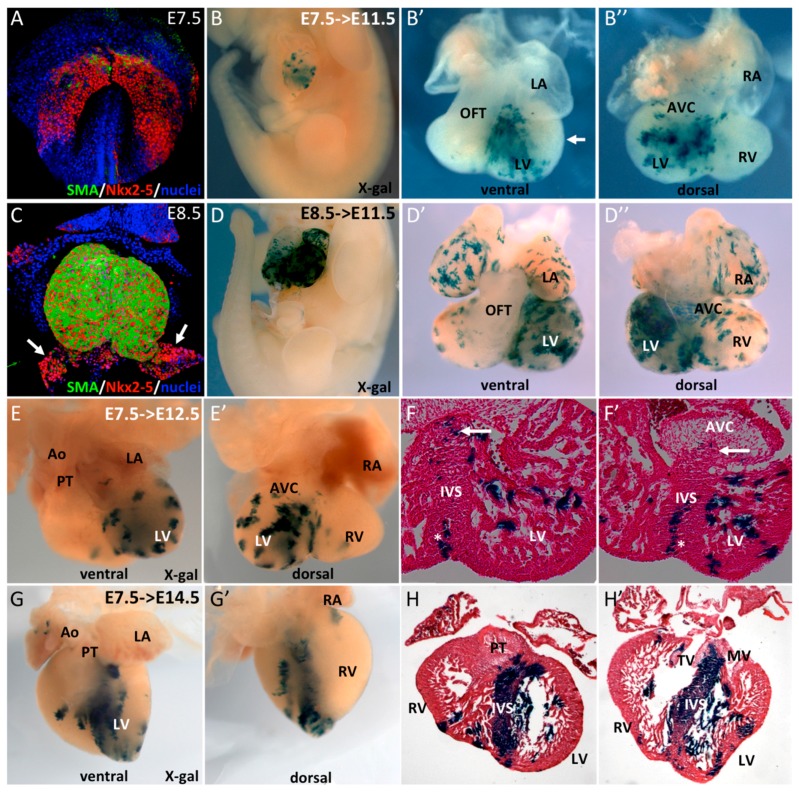
Early SMA+ cardiomyocytes contribute to the interventricular septum. (**A**–**C**) Whole-mount immunofluorescence reveals early expression of SMA in Nkx2-5+ cells. At E7.5, SMA is expressed in a subpopulation of cells in the cardiac crescent and, at E8.5, in the early heart tube with the exception of the venous pole (arrows). (**B**–**D**) Whole-mount X-gal staining of E11.5 *Sma^Cre/+^::R26R^LacZ/+^* embryos after tamoxifen injection at E7.5 (B) or E8.5 (**D**). (**B’**,**B’’**) ventral and dorsal views of an E11.5 heart showing X-gal labeling in the left ventricle (LV) and interventricular septum (IVS). No labeling was observed in left ventricular free wall myocardium (arrow). (**D’**,**D’’**) ventral and dorsal view of E11.5 heart with X-gal labeling throughout the heart with the exception of the OFT. Ventral (**E**,**F**) and dorsal (**E’**,**F’**) whole-mount and serial sections of E12.5 *Sma^Cre/+^::R26R^LacZ/+^* embryos after tamoxifen injection at E7.5. X-gal labeled cells are present in the LV and IVS with sparse positive cells at the upper part of the septum (arrows), in contrast to compact vertical clusters at the base of the IVS (stars). Ventral (**G**,**H**) and dorsal (**G’**,**H’**) whole-mount and serial sections of an E14.5 *Sma^Cre/+^::R26R^LacZ/+^* embryo after tamoxifen injection at E7.5. X-gal labeling is intense in the IVS and few clusters are observed in the LV.

Using whole-mount immunofluorescence, SMA expression was detected at E7.5 in the anterior medioventral position of the cardiac crescent expressing the transcription factor Nkx2-5 (Figure 1A). At E8.5, SMA is uniformly present in the primary heart tube while Nkx2-5 expression is also observed in the sinus venosus region (Figure 1C). SMA+ early cardiomyocytes thus appear to identify differentiated myocardial cells within the primary heart tube.

To establish the fate of these early cardiomyocytes during heart development we performed a genetic tracing of SMA+ cells. *Sma^CreER2/+^* mice were crossed with mice carrying a *R262R^LacZ/+^* conditional lineage reporter and Cre was activated upon 4-hydroxytamoxifen injections at early stages of development. After Cre activation at E7.5 or E8.5, β-galactosidase expression was observed only in the heart of E11.5 embryos (Figure 1B,D), highlighting the cardiac restricted expression of SMA at early developmental stages. Closer examination of these hearts revealed different β-galactosidase staining patterns between these two timepoints. Cre induction at E7.5 results in labeling of the interventricular region and part of the left ventricle, both derivatives of the FHF, while both atria, the outflow tract (OFT), the right ventricle, and atrioventricular canal are devoid of β-gal+ cells. (Figure 1B’,B’’). No labeling was observed in the outer curvature of the left ventricle (arrow in Figure 1B’). Cre induction at E8.5, in contrast, results in β-gal+ cells in all cardiac compartments with the exception of the outflow tract and the sinus venosus (Figure 1D’,D’’), two regions derived from the SHF, and the last cells to enter the heart tube [23]. These results highlight the progressive activation of *Sma* as different compartments of the heart form, revealing consecutive formation of the interventricular region and the left ventricle, then the atria, the right ventricle and the atrioventricular canal and finally the OFT and SV.

The contribution of early SMA+ cells induced at E7.5 was investigated at later stages of heart development. At E12.5, a comparable staining with that at E11.5 (Figure 1B’,B’’) was observed (Figure 1E,E’). Sections of these hearts show β-gal staining in the interventricular septum (IVS) and both compact and trabecular zones of the LV (Figure 1F,F’). SMA-derived cells are found in the entire IVS, however the upper part of the septum contained fewer and more sparse β-gal+ cells compared to the apical part of the IVS. At later stages, these SMA+ cardiac progenitors contribute predominantly to the formation of the IVS and trabeculae of the LV; only very few β-gal+ cells are observed in the compact myocardium in the apex of the LV (Figure 1G,H). Together, these data show that SMA+ cardiac cells at E7.5 form the primary heart tube and contribute at later stages to the formation of the interventricular septum and left ventricular trabeculae, consistent with FHF contributions to the developing heart.

### 3.2. SMA+ Early Cardiac Cells Contribute to the Ventricular Conduction System

**Figure 2:**
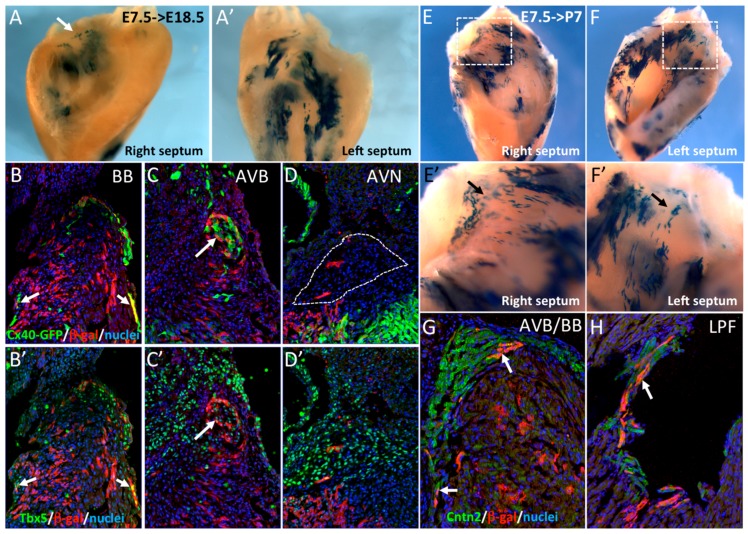
Early SMA+ cardiomyocytes contribute to the central ventricular conduction system. (**A**,**A’**) Whole-mount X-gal staining of the right and left sides of an E18.5 dissected septum after Cre induction at E7.5. Labeled cells are more abundant on the left side and sparse labeling is observed at the crest of the septum (arrow). (**B**–**D**) Immunofluorescence on sections of E18.5 hearts from *Cx40^GFP/+^::Sma^Cre/+^::R26R^lacZ/+^* mice after Cre activation at E7.5. SMA-derived cells are detected in the right and left bundle branches (BB) and are positive for Cx40-GFP (**B**) and Tbx5 (**B’**) (arrows). SMA+ cells participate to the AVB, that is also positive for Cx40-GFP (**C**) and Tbx5 (**C’**), but rarely to the AVN which is negative for Cx40-GFP (**D**) but positive for Tbx5 (**D’**). (**E**,**F**) Whole-mount X-gal staining of the right and left side of a P7 dissected septum after Cre induction at E7.5. High magnification of the septum crest (**E’**,**F’**) shows scattered X-gal positive cells in the region of the forming central VCS. (**G**,**H**) Immunofluorescence on sections of a P7 *Sma^Cre/+^::R26R^lacZ/+^* heart after Cre activation at E7.5 showing co-localization of SMA-derived cells and Contactin-2 (Cntn2) in the AVB (**G**) and left Purkinje fibers (**H**), indicated by arrows.

In order to investigate the cell lineage contribution of these SMA+ early cardiac cells to the ventricular conduction system (VCS), *R262R^LacZ/+^::Sma^CreER2/+^* mice were crossed with *Cx40^GFP/+^* mice expressing GFP specifically in the VCS at fetal and adult stages [19]. Cre activation was performed by tamoxifen injection at E7.5. Hearts recovered at E18.5 or P7 were dissected to isolate the IVS before X-gal staining. At E18.5, β-gal labeling was mainly detected on the left side of the septum (Figure 2A’) and at the crest of the septum in the region of the AVB (arrow in Figure 2A). To further investigate the contribution of these cells to the VCS, we performed immunofluorescence on sections using anti-β-gal and Tbx5 antibodies. Co-expression of Cx40-GFP or Tbx5 with β-gal is observed in both bundle branches (BB) and the AVB (Figure 2B,B’,C,C’). Only very few β-gal+ cells were detected in the AVN (Figure 2D), which is known to derive from the AVC [24]. These results were confirmed in postnatal hearts (P7), by which stage the definitive VCS is established [25]. Extensive labeling was observed on both sides of the IVS (Figure 2E,F) and β-gal+ cells were observed along the crest of the IVS and both sides of the septum (Figure 2E’,F’). This distribution of β-gal+ cells is characteristic of the pattern of the VCS. To confirm that early SMA+ cardiomyocytes contribute to the VCS, immunofluorescence with Contactin-2 and β-gal antibodies was performed and revealed double positive cells in the AVB, BB, and left Purkinje Fibers (Figure 2G,H). These results demonstrate that early SMA+ cardiomyocytes contribute to the formation of the VCS but not to the AVN.

### 3.3. The Central Ventricular Conduction System Lineage Segregates from SMA+ Early Cardiomyocytes

**Figure 3:**
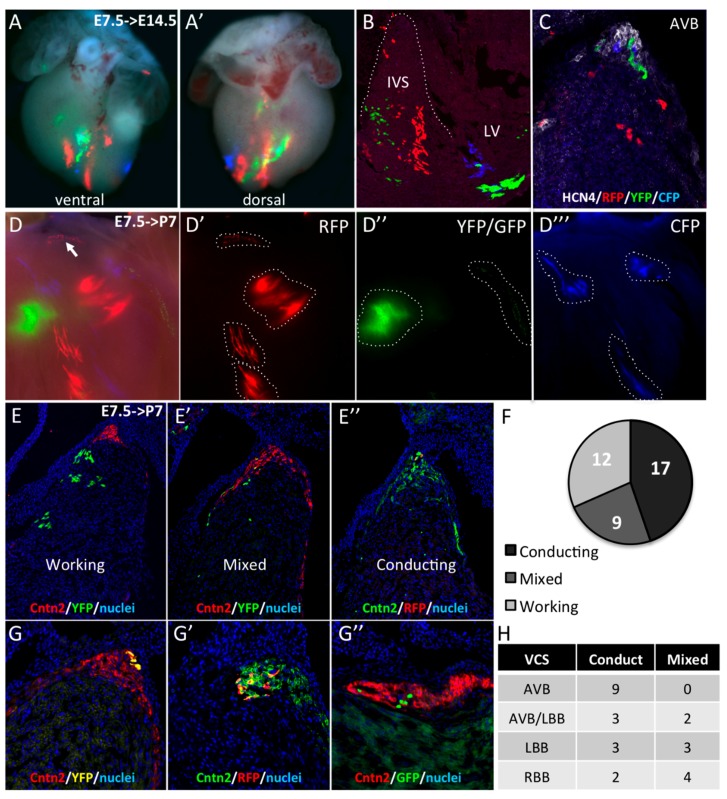
Early segregation of the central ventricular conduction system lineage in SMA+ cardiomyocytes. (**A**,**A’**) Whole-mount ventral and dorsal views of an E14.5 *Sma^Cre/+^::R26R^confetti/+^* heart with multicolor clusters induced by tamoxifen injection at E7.5. The four fluorescent reporters (GFP, YFP, RFP, and CFP) form independent clusters distributed side by side along the vertical axis in the apical interventricular region. (**B**) Section of an E14.5 *Sma^Cre/+^::R26R^confetti/+^* embryonic heart showing large vertical compact multicolor clones in the apical region of the IVS and scattered labeled cells in the upper part of the septum. (**C**) Hcn4 immunofluorescence on section of E14.5 *Sma^Cre/+^::R26R^confetti/+^* heart showing a heterogeneous contribution of labeled cells to the AVB and BB. (**D**) Example of clonal labeling in a P7 *Sma^Cre/+^::R26R^confetti/+^* heart with a small number of labeled clusters of which one RFP+ cluster is localized at the crest of the septum (arrow). Individual fluorescent reporters are presented in **D’** (RFP), **D’’** (GFP/YFP) and **D’’’** (CFP). (**E**,**G**) Cntn2 immunofluorescence on sections from P7 *Sma^Cre/+^::R26R^confetti/+^* hearts to classify the nature these clusters. All cells in working clusters are negative for Cntn2 (**E**), few are positive in mixed clusters (**E’**) and all are positive for Cntn2 in conducting clusters (**E’’**). The distribution of these clusters is presented in the graph in the right panel (**F**). (**G**,**G’**,**G”**) Sections through independent conducting clones of different colors (YFP in **G**; RFP in **G’** or GFP in **G’’**) showing co-labeling with Cntn2 in the AVB. (**H**) Table showing the distribution of mixed and conducting clones in different compartments of the central VCS.

Working and conducting cardiomyocytes are derived from common progentior cells [25], although the time of working and VCS lineage divergence is unknown. In order to investigate whether E7.5 SMA+ cardiomyocytes giving rise the VCS have already segregated from cells giving rise to working myocardium, *Sma^CreER2/+^* mice were crossed with *R26R^confetti/+^* lineage reporter mice to generate multicolor clones [15]. R26R-confetti mice possess four fluorescent reporter genes (RFP, CFP, YFP, and GFP) that are randomly activated upon Cre deletion [20]. We performed pulse activation of Cre recombinase by injecting 4-hydroxytamoxifen into pregnant females at E7.5. At E14.5, multicolor clusters were detected in the interventricular septum (Figure 3A,B). Observations from a large number of hearts with multicolor clones revealed two types of clusters. In the apical two-thirds of the IVS, large and elongated unicolor clones are juxtaposed and distributed among the vertical apex to base axis (Figure 3A,B). In contrast, in the upper part of the IVS, the distribution of labeled cells is more heterogenous with sparse randomly-distributed cells. To investigate the contribution of these labeled cells to the AVB and BB, Hcn4 immunofluorescence was performed on sections. Few labeled cells were found in the AVB and the clonal relationship between independent cells of the same color in the AVB was difficult to establish because of the dispersed distribution (Figure 3C). In order to perform a clonal lineage analysis of the SMA+ early cardiomyocytes, we used a low dose of 4-hydroxytamoxifen to obtain between 1 and 20 multicolor clusters per heart. First, we observed the distribution of these clusters in whole-mount dissected IVS from postnatal day 7 (P7) hearts. Individual clusters of uniform color could be readily distinguished on the left side of the IVS (Figure 3D), therefore cells from each cluster are considered to be clonally related. Large and compact clones are found in the IVS while a RFP+ clone of sparse cells can be seen in the upper part of the septum where the AVB develops (Figure 3D,D’). As all these sparse cells express the same color, they are considered to be part of the same clone. In total, we obtained a collection of 190 clusters from which 26 are in the central VCS defined by Contactin-2 immunostaining on sections. In the upper part of the IVS, three types of clones could be identified in relation to their contribution or not to the AV conduction system: no labeled cells in the AV conduction system (working, Figure 3E); few cells in the central VCS (Mixed, Figure 3E’) or all cells in the central VCS (Conducting, Figure 3E’’). In mixed clones, cells are distributed along both sides of the IVS (Figure 3E’). In contrast to working clones, cells in conducting (Figure 3E’’) and mixed clones show a dispersed distribution, suggesting a different mode of growth compared to compact working clones. The number of exclusively conducting clones is twice the number of mixed clones (Figure 3G). Finally, we studied the distribution of the mixed and conducting clones in different components of the central VCS (Figure 3H). The nine clones identified in the AVB are entirely Contactin-2+ (Figure 3F) while only half of the clones covering the BBs are exclusively conducting. Early *Sma* expressing cardiomyocytes therefore include cells exclusively fated to give rise to the AVB, suggesting early segregation of central VCS lineages. In the case of the BB component, lineage segregation is still ongoing at E7.5. This result is consistent with the existence of a dedicated population of central VCS precursors expressing *Sma* at the early heart tube stage.

### 3.4. SMA-Derived Early Cardiomyocytes form the Primary Heart Tube

**Figure 4:**
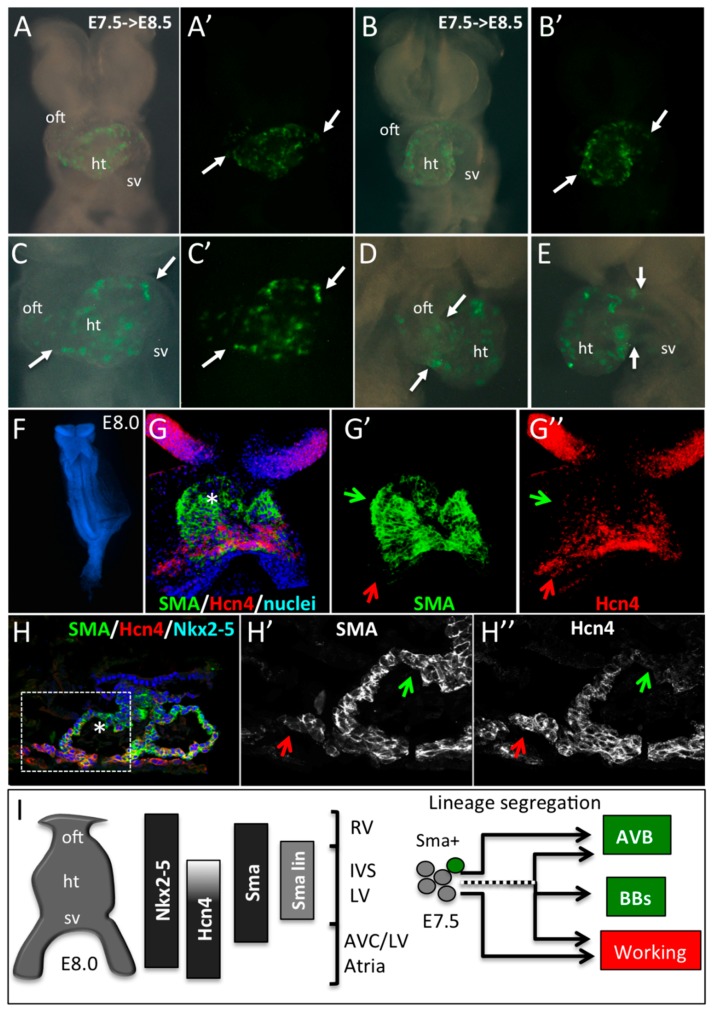
Early SMA+ cardiomyocytes form the primary heart tube. (**A**–**E**) Whole-mount bright field or YFP fluorescence (**A’**–**C’**) of E8.5 *Sma^Cre/+^::R26R^YFP/+^* embryonic hearts induced by tamoxifen injection at E7.5. Ventral (**A**–**C**), right lateral (**D**) or left lateral (**E**) views show the presence of YFP+ cells in the heart tube only (ht). Arrows indicate the limit of YFP expression in the heart tube and regions with no labeling such as the outflow tract (oft) and sinus venosus (sv). (**F**–**H**) Immunofluorescence on whole-mount (**G**,**G’**,**G’’**) and sections (**H**,**H’**,**H’’**) of E8.0 embryos reveals a co-expression of SMA and Hcn4 in the lower part and a SMA+/Hcn4- expression domain in the upper part of the heart tube (star in **G**,**H**). An Hcn4 expression domain is observed at the venous pole (red arrows). Green arrows indicate the presumptive position of the early SMA+ cardiomyocytes in E8.0 heart tube. (**I**) Schematic representation of the expression domains of Nkx2-5, Hcn4, SMA and E7.5 SMA+ lineage (SMA lin) in the linear heart tube of an E8.0 mouse embryo. The compartments of the heart derived from different subdomains of the early heart tube are shown: RV, Right ventricle; IVS, Interventricular septum; LV, Left ventricle; AVC, Atrioventricular canal, and Atria. Our data suggest that precursors of the central VCS are included in the SMA+/Hcn4- region of the heart tube. While some early SMA+ cardiomycytes have already segregated to the conduction lineage to form the AVB, others, including cells giving rise to the bundle branches (BB), segregate later in embryonic development.

To study the contribution of these early SMA+ cardiomyocytes to the primary heart tube, we performed a genetic tracing by crossing *Sma^CreER2/+^* mice with *R26R^YFP/+^*. 4OH-tamoxifen was injected to pregnant females at E7.5 and embryos were recovered 20 h later at E8.5. YFP+ cells are detected in the central region of the heart tube but not at the cardiac poles (Figure 4A–E). These data are consistent with the conclusion that the early heart tube gives rise predominantly to the IVS and only part of the LV.

To better understand where the precursors of the central VCS are localized in the early heart tube, we compared the expression of Nkx2-5, HCN4, and SMA in embryos at E8.0. Hcn4 expression is dynamic during cardiac development and temporally controlled lineage tracing of HCN4-derived cells reveals that their contribution to the AVB is low at E7.5 and high at E8.5 [14]. These results suggest that HCN4 expression is low in AVB precursors in the hours following tamoxifen injection at E7.5. At the 3–5 somites stage, SMA is detected in the heart tube while HCN4 expression is more intense around the sinus venosus and absent at the arterial pole (Figure 4F,G). Immunofluorescence on sections shows co-labeling of SMA with Nkx2-5 in cardiomyocytes within the heart tube but not in progenitor cells at the arterial and venous poles (Figure 4H). Taking into account the lineage tracing of SMA and HCN4 at E7.5 and E8.5, and their respective expression profiles, we conclude that AVB precursors localize in the anterior region of the heart tube at E8.0 (Figure 4I).

## 4. Discussion and Conclusions

Cre-mediated genetic labeling has accelerated our understanding of how different cell lineages contribute to the heart. It is now well established that the primary heart tube forms the left ventricle while the other cardiac compartments are added during subsequent development at the venous and arterial poles [9]. This conclusion is based on the analysis of mouse mutants and genetic tracing showing that the LIM homeodomain transcription factor Isl1 is selectively expressed in SHF cells, which will form the outflow tract, right ventricle, right atria, and a portion of cells of the left ventricle and left atria. More recently, studies using inducible *HCN4-CreERT2* and *Tbx5-CreERT2* mice have shown that early progenitor cells labeled at E7.0 or E6.5, respectively, delineate FHF derivatives such as the left ventricle and both atria [13,14,26]. The results of our genetic Cre lineage analysis of early Sma+ cardiomyocytes are consistent with a progressive contribution of cells to the heart, with the earliest Sma+ cells giving rise to the interventricular septum. This conclusion is in agreement with data from early fate mapping of the cardiac tube in chicks showing that the primordium of the interventricular septum appears at the site of ventral fusion of bilateral cardiac primordia [27,28]. After Cre activation at E7.5, Sma+ cells give rise exclusively to the IVS and left ventricular apex. With Cre activation one day later, labeled cells were observed in all cardiac compartments, with the exception of the OFT region and sinus venosus which contribute later to the heart from the SHF. The boundary between the IVS and LV has been already documented using a *Tbx2-Cre* mouse line suggesting that the LV free wall myocardium derives from progressive addition of cells from the AVC [24]. The transcription factor Tbx2 is expressed early in the AVC and lineage tracing of these cells shows that they contribute to a large part of the left ventricular free wall, but not to the septum or LV apex. Similarly, Cre activation at gastrulation (E6.0) and early crescent stage (E7.0) of Hcn4+/FHF cells demonstrated a demarcation between labeling events in the IVS and the LV with the LV but not IVS being labeled when tamoxifen was injected at E7.0 [13]. It has been described that Hcn4 expression decreases as FHF progenitors are incorporated and that at the early heart tube stage, HCN4 positive cells are localized close to the venous pole [13]. Genetic tracing lineage analysis of Tbx5 labeled at E6.5 confirmed that early FHF cells give rise to the IVS and LV [26]. As the expression patterns of Hcn4 and Tbx5 are largely overlapping in the presumptive FHF [13], these data suggest that the IVS is derived from FHF progenitor cells that have already started to downregulate Hcn4 expression at the early heart tube stage. In contrast to Isl1, Hcn4, or Tbx2, SMA is stably expressed in early cardiomyocytes and is not restricted to specific cardiac compartments, so our data directly reflect the time of differentiation of these cells as they incorporate to the heart tube. Short-term lineage tracing of Sma+ cardiomyocytes shows that these cells contribute to the early heart tube but not to myocardium at the cardiac poles. In summary, the first Sma+ cardiomyocytes giving rise to the IVS are presumably negative for Tbx2 and Hcn4 and are localized in the anterior part of the early heart tube (Figure 4I).

The contribution of FHF and SHF progenitors to the development of the central VCS was previously studied using several genetic approaches [10]. From these studies, FHF cells were shown to contribute exclusively to the LBB and Left PF while SHF cells contribute to the SAN and right Purkinje fibers. However, a dual contribution of cells from both heart fields has been proposed during the formation of the AVN, AVB, and RBB [12,14]. In our SMA genetic tracing experiments, we showed that the early cardiomyocytes contributing to the IVS also give rise to the AVB, LBB, and a small contribution to the RBB but almost none to the AVN. This result suggests that precursors of the central VCS are included in these early cardiomyocytes and that the formation of the definitive central VCS necessitates the contribution of cells from the AVC to form the AVN and SHF to form the AVB and RBB, as demonstrated by Tbx2 and Isl1 genetic tracing experiments [24,29]. Together, these data suggest that precursors of the VCS originate from both heart fields.

Previous studies have suggested that a population of early cardiomyocytes expressing GlN2 in human embryonic hearts localized at the inner curvature forms a primary ring at the interventricular foramen corresponding to the central VCS [5]. However, the lineage relationship between the embryonic primary ring and the definitive central VCS was unclear. Our clonal labeling of early cardiac cells highlights the early lineage segregation to the central VCS before formation of the primary ring. These data suggest that early cardiomyocytes are assigned to a conductive fate in the heart and maintain this function until postnatal stages. This is particularly true for the AVB and reinforces our earlier identification of a clonal boundary between the AVB and the upper septum [12]. However, in our previous retrospective clonal analysis, it was not possible to date this lineage segregation. The comparison of expression patterns between Hcn4 and SMA at E8.0 suggests that these precursors are included in the anterior part of the primary heart tube that gives rise to the IVS.

Using *Hcn4CreERT2* mice, tamoxifen injection at E7.5 labeled FHF derivatives including the LBB but only partially labeled the AVB, which is strongly labeled after tamoxifen injection at E8.5, suggesting that the AVB differentiates after the LBB [14]. However, in our prospective clonal analysis of SMA+ early cardiomyocytes, we observe segregation of the AVB lineage at the early heart tube stage. The presence of mixed clones with cells in the conduction system and in the IVS suggests that the separation between these two lineages is not complete at this early stage of development for the right and left BB. These data are in accordance with our previous retrospective clonal analysis indicating the existence of early common progenitors for conductive and working cardiomyocytes [12,25]. Our new data using SMA prospective clonal analysis suggests that the AVB is specified earlier than the BB or during a more restricted time window. The discrepancy between our conclusions and those drawn from the Hcn4 fate mapping analysis is likely to result from the different expression patterns of these genes and the experimental approach. Indeed, the expression pattern of *Hcn4* is particularly complex during cardiac development with an early expression in FHF progenitors and, from E16.5, a restriction to the CCS [13,14]. Moreover, clonal analysis is a strategy more appropriate to distinguish lineage segregation. Together, these data demonstrate that the segregation of the AVB lineage takes place as early as the heart tube stage in early Sma+ cardiomyocytes, in which re-expression or de novo expression of Hcn4 has not yet occurred (Figure 4I). This conclusion suggests that segregation of the AVB lineage takes place slightly before the onset of the first cardiac contractions at the 5-somite stage [30]. Potentially, distinct AVB progenitor cells may exist even prior to myocardial differentiation. Overall, our data suggest a progressive segregation of the central VCS derived from early cardiomyocytes starting with the AVB, followed by cells forming the right and left BB. The presence of mixed clones shows that growth of the definitive central VCS requires the later addition of cells from other sources of progenitors. Indeed the pattern of these clones at the subendocardial layer along a vertical axis suggests an extension of the septum crest on both sides accompanying the growth of the IVS as has been described for the development of the BB [31]. It remains to be seen if lineage segregation of these cells occurs in the early heart or if adjacent cardiomyocytes contribute to the central VCS during later heart development.

Further understanding of the molecular and cellular basis of these lineage segregation events, and more precisely, the early formation of specialized conducting cardiomyocytes from common precursors with working myocytes will be of great relevance to conduction diseases and for regenerative strategies to replace the conduction system by bio-artificial pacemakers.
